# Case Report: When syphilis imitates cancer: a case of misdiagnosed cutaneous T-cell lymphoma

**DOI:** 10.3389/fonc.2026.1730564

**Published:** 2026-03-16

**Authors:** Ugo Giordano, Jacek Kwiatkowski, Krzysztof Zduniak, Monika Mordak-Domagała, Zuzanna Dybko, Jarosław Dybko

**Affiliations:** 1 Department and Clinic of Endocrinology and Internal Medicine, Wroclaw Clinical Hospital, Wroclaw, Poland; 2 Department of Hematology and Cellular Transplantation, Lower Silesian Oncology Center, Wroclaw, Poland; 3 Faculty of Medicine, Wroclaw Medical University, Wrocław, Poland; 4 Department of Oncology and Hematology, Faculty of Medicine, Wroclaw University of Science and Technology, Wroclaw, Poland

**Keywords:** CTCL (cutaneous T-cell lymphoma), FDG-PET-CT, hematological malignancy, misdiagnosis, syphilis

## Abstract

Syphilis is an infectious disease caused by Treponema pallidum, which is regarded as one of the most significant imitators in daily clinical practice. Its manifestations are various, comprising numerous autoimmune, inflammatory, and neoplastic disorders. Here we present a case of a 27-year-old male initially suspected of cutaneous T-cell lymphoma (CTCL) based on lymphadenopathy, systemic weight loss, ulcerative cutaneous lesions, and fluorodeoxyglucose positron emission tomography-computed tomography (FDG-PET-CT) findings of hypermetabolic nodes above and below the diaphragm. Histopathological analysis suggested pleomorphic lymphoid proliferation, while imaging supported the suspicion of malignancy. However, subsequent biopsies revealed plasmacytic and lymphocytic infiltration without clonality, and treponemal serologies confirmed secondary syphilis. The patient was referred for anti-treponemal therapy resulting in the resolution of the initial symptoms. This case presents how syphilis can closely imitate hematologic malignancy, particularly CTCL, both clinically and histopathologically. We also review and prepare a summary of published reports of syphilis mimicking malignancies and propose a structured diagnostic algorithm. We believe that the early inclusion of syphilis in differential diagnosis is critical to avoid misdiagnosis and delays in initiating appropriate therapy.

## Introduction

Syphilis, caused by Treponema pallidum, has long been known to mimic numerous diseases, owing to its complex presentations across dermatologic, neurologic, cardiovascular, and hematologic systems (1). In its secondary stage, syphilis frequently manifests with mucocutaneous lesions and generalized lymphadenopathy, but in atypical cases, its features may overlap with malignant or autoimmune diseases (2, 3). This diagnostic challenge is posed by the lack of specificity of modern imaging techniques, such as FDG-PET-CT, which may show hypermetabolic lymph nodes in both malignant lymphoma and infectious diseases (4, 5).

Numerous unusual presentations of syphilis have been reported based on available clinical data, including pulmonary nodules, gastric masses, cervical ulcerations, skin rash, and widespread lymphadenopathy, often leading to an initial misdiagnosis of lymphoma or malignancy (5–11), as presented in **Table 1**. In immunocompromised individuals such as those with HIV, syphilis may histologically and clinically resemble cutaneous T-cell lymphomas (CTCL) (7).

**Table 1:** Reported cases of syphilis misdiagnosed as hematologic or non-hematologic malignancies.

First author (Year)	Clinical presentation	Initial suspected diagnosis	Diagnostic pitfall	Results leading to syphilis diagnosis
Ohta et al. (4)	Patient presenting with chest pain, stomatitis, rash, diffuse lymphadenopathy, and pulmonary nodules	Metastatic lung carcinoma or lymphoma	Increased FDG uptake in mediastinal and axillary lymph nodes and pulmonary nodules on PET-CT; lymph node biopsy showing nonspecific findings	Reactive RPR/TPHA serology; clinical improvement and radiologic regression of pulmonary nodules following antibiotic therapy
Cerchione et al. (6)	Generalized lymphadenopathy accompanied by fever, weight loss, cutaneous rash, hepatosplenomegaly, and night sweats	Non-Hodgkin lymphoma	FDG-PET-CT showing multiple hypermetabolic lymph nodes; cytology revealing reactive hyperplasia; late-onset rash misinterpreted as viral exanthem; negative flow cytometry for B/T-cell clonality	Treponemal serology positive upon repeat testing; rapid resolution following penicillin therapy; history of high-risk sexual exposure
Yamashita et al. (7)	HIV-positive patient with ulcerated cutaneous lesions, fever, cephalalgia, and myalgia, without lymphadenopathy	CTCL	Histopathology showing CD8^+^ T-cell infiltrates consistent with CTCL; absence of initial recognition of reactive pattern	Lack of TCR gene rearrangement; positive treponemal tests; spirochetes visualized microscopically
Lan et al. (5)	Epigastric pain, weight loss, and an endoscopically visualized gastric mass	Gastric adenocarcinoma or lymphoma	Endoscopic biopsy showing chronic gastritis with ulceration; CE-CT and FDG-PET-CT suggesting malignancy	Immunohistochemical detection of T. pallidum; positive serology; absence of neoplastic cells; relevant sexual history
Salah et al. (9) – Case 1	Widespread erythematous plaques on the skin	Primary cutaneous marginal zone lymphoma	Skin biopsy showing lymphocytic infiltrate with CD3^+^ T-cells and CD20^+^ B-cells; B-cells expressing BCL2 and BCL6 with a predominance of kappa-positive plasma cells	Positive syphilis serology; immunohistochemistry confirming spirochetes; flow cytometry negative for lymphoma
Salah et al. (9) – Case 2	Severe gastritis and esophageal candidiasis preceding disseminated papular eruption	Drug-induced eruption following antifungal treatment	Biopsy showing atypical CD3^+^, CD8^+^, TCRβF1^+^, CD7^+^ T-cells with immunoreactivity for TIA-1 and granzyme B; normal bone marrow morphology and flow cytometry	Immunohistochemical detection of spirochetes; positive serologic testing for syphilis
Hodak et al. (11)	Nodular cutaneous eruption with dense plasma-cell infiltrates	Cutaneous lymphoma	Histopathologic interpretation suggesting lymphoma; failure to recognize reactive plasmacytosis	Positive serologic testing for syphilis; dark-field microscopy confirming spirochetes; complete clinical remission following penicillin
Komeno et al. (10)	Bilateral cervical lymphadenopathy, pulmonary lesion, and periportal lymph node enlargement	Malignant lymphoma	Lymph node biopsy demonstrating fibrotic adipose tissue infiltrated by lymphocytes with mildly enlarged nuclei	Positive treponemal serology; resolution after penicillin therapy; history of oral sexual contact
Maci et al. (8)	HIV-positive patient under antiretroviral therapy with fever, multiple lymphadenopathies, and pulmonary consolidation	Hematologic malignancy	Lymph node biopsy showing architectural disruption with granulomatous reaction and multinucleated giant cells; negative anti-treponemal immunoreactivity	Positive serologic tests; clinical and radiologic resolution post-penicillin treatment; regression of pulmonary findings on FDG-PET-CT

FDG-PET-CT, fluorodeoxyglucose positron emission tomography-computed tomography; CTCL, cutaneous T-cell lymphoma; HIV, human immunodeficiency virus; CE-CT, contrast-enhanced computed tomography; RPR, rapid plasma reagin test; TPHA, treponema pallidum hemagglutination assay.

Given its rising incidence globally, especially among at-risk populations, syphilis should remain an essential part of differential diagnoses when assessing patients with systemic symptoms, lymphadenopathy, and cutaneous lesions suspicious for malignancy (2, 12). Herein, we present a patient initially suspected of CTCL, ultimately diagnosed with secondary syphilis, provide a review of similar cases from the literature, and propose a structurized diagnostic algorithm based on our experience and previously published scientific reports.

## Case report

**Figure 1:**
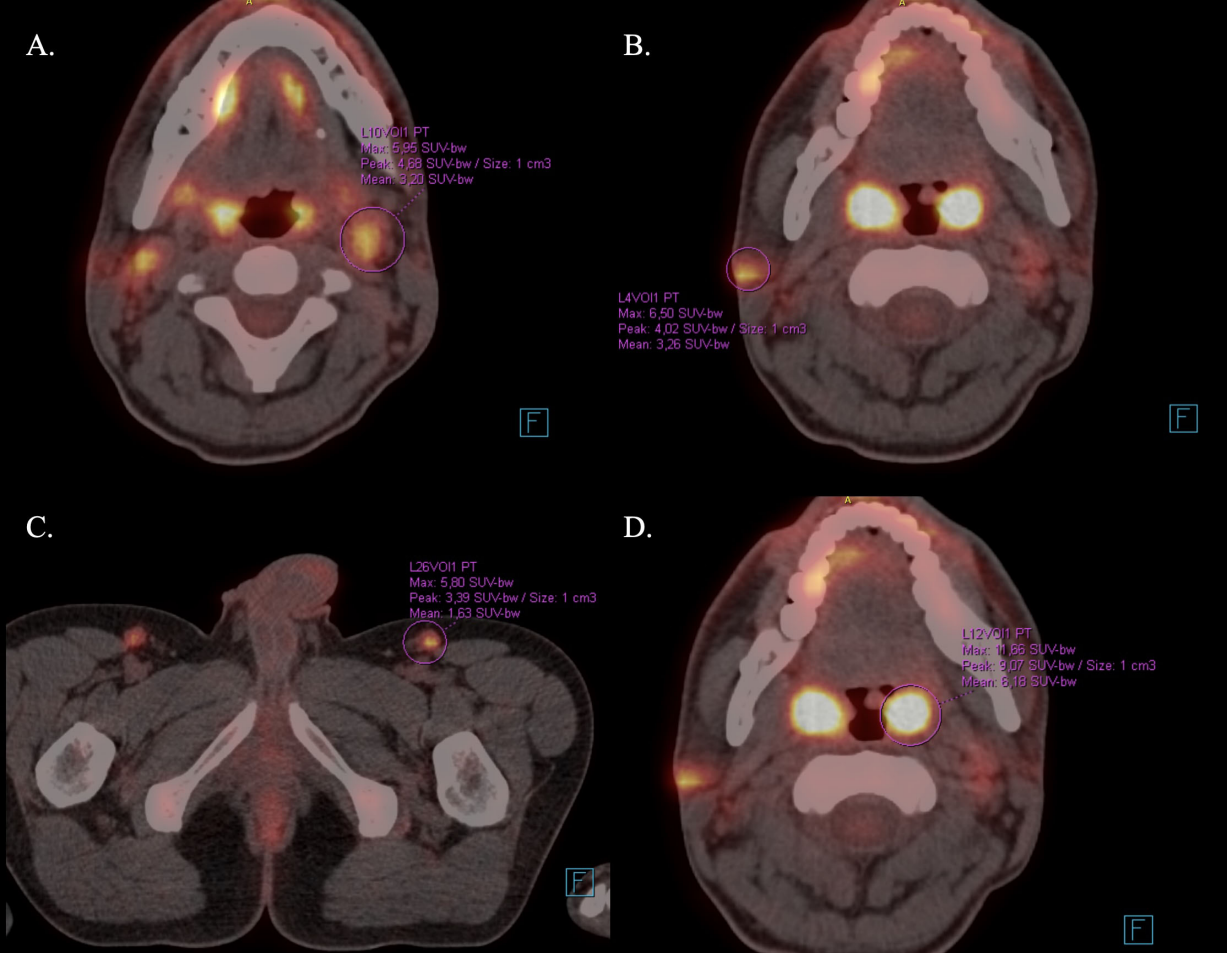
FDG-PET-CT demonstrating diffuse hypermetabolic lymphadenopathy above and below the diaphragm, including bilateral cervical **(A)**, axillary, inguinal **(C)**, and para-aortic lymph nodes (SUVmax up to 6.8), with symmetric tonsillar uptake **(D)** (SUVmax 11.7) and focal FDG uptake in the left vocal fold (SUVmax 8.2) and skin **(B)** (SUVmax 6.5). No structural pulmonary lesions were identified. In conjunction with the available results back then, the imaging pattern initially raised strong suspicion of lymphoproliferative disorder.

**Figure 2:**
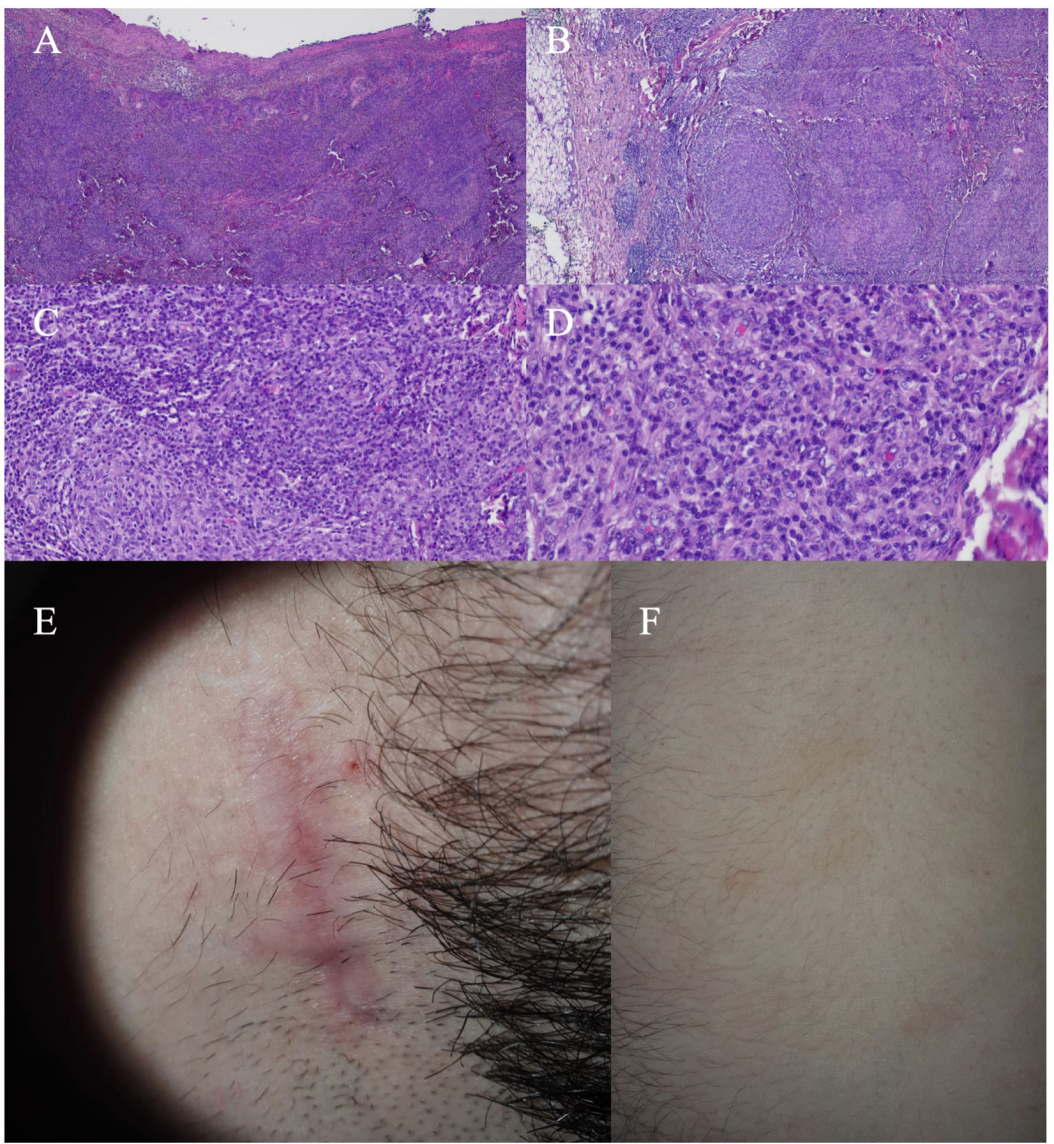
Histopathological examination of the ulcerated skin from October 2022 (**(A–D)** HE x20, x40, x200, x400, respectively). Histologically skin lesions presented as ulcerated tumors, composed mostly of ill-defined non-necrotizing granulomas and numerous mature plasma cells, which were polytypic on kappa/lambda immunohistochemistry. Mature lymphocytes, predominantly T-cells, were relatively sparse. There were some dispersed immunoblasts. Diagnosis of reactive, inflammatory lesions was rendered with suggestion of infectious disease. Ulceration in the right submandibular space in the scar after biopsy **(E)**. Disseminated lesions on the skin of the chest and abdomen **(F)**.

A 27-year-old male suffering from ankylosing spondylitis (HLA-B27 positive) was suspected of being affected by CTCL. The initial manifestation in June 2022 included progressive right cervical lymphadenopathy with a non-healing ulceration of the overlying skin. Also, the patient experienced an unintentional weight loss of 8 kg within 1.5 months (from 80 kg to 72 kg) without night sweats or fever. The patient did not report any recent travel history or known exposure to tuberculosis. Sexual history did not reveal any specific exposure disclosed at the time of initial evaluation. Laboratory tests revealed a mild, new-onset anemia (drop of hemoglobin concentration from 16 g/dL to 12.8 g/dL (N: 14–18 g/dL) in the observation period). In September 2022, an excisional biopsy of head and neck lesions and involved skin revealed an infiltration of pleomorphic CTCL (CD3 +++,CD20 +, Ki67 + in 10% of cells, Melan A -, HMB-45 +/-, MITF -, SOX10 -). At that time, a fluorodeoxyglucose PET-CT scan showed active proliferative disease involving lymph nodes above and below the diaphragm with cutaneous involvement, as presented in **Figure 1**. Additional tests were carried out in October 2022, including:

- Biopsy of the ulcerated skin - abundant infiltration of plasmocytes and lymphocytes in the dermis proper without neoplastic lesions (**Figures 2A–D**);
- Bone marrow biopsy - medium-density marrow, without the presence of abnormal cells, with changes in granulocytes and platelets consistent with vitamin B12 deficiency;
- Bone marrow cells flow cytometry - no evidence of abnormal clonal T-lymphocytes;
- Cytogenetics - no detectable TCRβ or TCRαδ gene rearrangements;
- Trephine biopsy - no presence of lymphoproliferative disease, consistent with reactive or inflammatory changes;
- Testing for infectious or autoimmune diseases (negative for HBV, HCV, HIV, HSV 1/2, VZV, HTLV I/II, EBV, CMV, anti-intrinsic factor, anti-gastric parietal cell antibodies, the ANA 4 test combining indirect immunofluorescence (IIF on HEp-2 cells) and immunoblot to detect antinuclear and anticytoplasmic antibodies identifying 16 specific antigens).

With these results, in March 2023, the patient was admitted to our ward in order to continue the diagnostic workup. He was in good general condition, with persistent left cervical and bilateral inguinal lymphadenopathy (lymph nodes up to 1.5 cm and 1 cm, respectively). Cutaneous involvement was present as infiltrative skin lesions including the scar after lymph node biopsy on the neck, chest, trunk, back, and scrotum, without pruritus or scaling (**Figures 2E, F**). Further testing revealed an IgG4 level within normal ranges with, however, a positive result of the Waaler-Rose, fluorescent treponemal antibody absorption (antibody titer 1:4000), treponema pallidum hemagglutination assay (antibody titer 1:10240), and venereal disease research laboratory (antibody titer 1:128) tests. Considering the positive treponema pallidum tests, and results of trephine and ulcerated skin biopsy (**Figures 2A–D**) suggesting the occurrence of an inflammatory process, a suspected syphilis diagnosis with skin involvement was made. The higher lymph node metabolic activity both above and below the diaphragm in the PET-CT scan from October 2022 was deemed secondary to the ongoing inflammatory process. As a consequence, in April 2023, the patient was referred to the Infectious Disease Department for treatment continuation. Overall, the clinical image including diffuse lymphadenopathy, cutaneous manifestations and reactive serology was most consistent with secondary syphilis rather than an early, latent infection. Therapy consisted of the administration of benzathine penicillin G 2.4 million units intramuscularly in a single dose, according to the current guidelines (13, 14). Clinical follow-up documented resolution of skin lesions and regression of lymphadenopathy within 12 weeks from treatment institution. Serologic response was monitored using non-treponemal titers including rapid plasma reagin (RPR) and VDRL, as recommended for secondary syphilis (13, 14), with re-evaluation carried out at 1, 3, 6 and 12 months after therapy. The resolution of clinical symptoms together with a ≥4-fold decline in non-treponemal titers (RPR and VDRL; 1:256, 1:128, 1:32, 1:4, 1:2 for RPR; 1:128, 1:64, 1:16, 1:4, 1:2 for VDRL; respectively at 0, 1, 3, 6, 12 months) at follow-up fulfilled serologic criteria of adequate treatment response and confirmed therapeutic effectiveness (13, 14). A table containing the summary of the course patient’s history is presented in **Table 2**.

**Table 2:** Timeline and course of the patient’s history.

Time point	Key event	Diagnostics	Key result/suspicion
June 2022	Lymphadenopathy, weight loss, ulcerative skin lesions	Physical exam; baseline laboratory testing	Suspicion of malignancy
September 2022	First histopathological evaluation	Excisional biopsy of head and neck lesions and involved skin	Infiltration of pleomorphic CTCL suspected
October 2022	Persistent symptoms	FDG-PET-CT	Hypermetabolic lymphadenopathy, lymphoma considered
October 2022	Second histopathological evaluation	Biopsy of the ulcerated skin - **Figure 2**	Reactive plasmacytic infiltrate; no clonality
October 2022	Continuation of diagnostic workup	Laboratory testing - infectious and autoimmune diseases; bone marrow and trephine biopsies; bone marrow flow cytometry; cytogenetics	No specific findings
March 2023	Admission to our ward; persistent lymphadenopathy, disseminated skin lesions	Syphilis diagnostic workup, IgG4	Positive Waaler-Rose, FTA-ABS, TPHA, VDRL tests; IgG4 within normal ranges
March 2023	Admission to Infectious Disease Department	RPR, VDRL	RPR - antibody titer 1:256, VDRL - antibody titer 1:128; benzathine penicillin G 2.4 million units IM in a single dose
April 2023	Serological response assessment and clinical evaluation at 1 month; lymphadenopathy and disseminated skin lesions reduction	RPR, VDRL	RPR - antibody titer 1:128, VDRL - antibody titer 1:64
June 2023	Serological response assessment and clinical evaluation at 3 months; minor lymphadenopathy present, disseminated skin lesions resolution reported in May 2023	RPR, VDRL	RPR - antibody titer 1:32, VDRL - antibody titer 1:16
September 2023	Serological response assessment and clinical evaluation at 6 months; resolution of all clinical symptoms	RPR, VDRL	RPR - antibody titer 1:4, VDRL - antibody titer 1:4
March 2024	Serological response assessment and clinical evaluation at 12 months	RPR, VDRL	RPR - antibody titer 1:2, VDRL - antibody titer 1:2

FDG-PET-CT, fluorodeoxyglucose positron emission tomography-computed tomography; CTCL, cutaneous T-cell lymphoma; IHC/flow, immunohistochemistry and flow cytometry; FTA-ABS, fluorescent treponemal antibody absorption; TPHA, Treponema pallidum hemagglutination assay; VDRL, Venereal Disease Research Laboratory test; RPR, rapid plasma regain; IM, intramuscularly. diagnostic workup at our ward started in March 2023.

## Patient perspective

The patient reported significant anxiety during the diagnostic process, primarily due to the initial concern for a malignant disease. The uncertainty surrounding the diagnosis was described as strongly stressing. Once the infectious etiology was identified and appropriate treatment was initiated, the patient experienced relief and expressed satisfaction with the rapid clinical improvement. The patient emphasized the importance of thorough diagnostic evaluation and improvement of quality of life and self-confidence after symptoms resolution.

## Discussion

Syphilis continues to pose significant diagnostic challenges due to its ability to mimic both hematologic and solid tumors. In our case, the initial clinical manifestation consisting of cervical lymphadenopathy, systemic weight loss, ulcerative cutaneous lesions, and PET-CT findings raised a strong suspicion of CTCL. Histopathology revealed pleomorphic lymphoid infiltration, while FDG-PET-CT demonstrated widespread hypermetabolic lymph nodes, further supporting this suspicion. However, repeated biopsies showed reactive plasmacytic infiltration without clonality, and subsequent treponemal serologies confirmed secondary syphilis. Although some patients may remain in a serofast state despite adequate therapy (13, 15), the significant reduction in non-treponemal titers and resolution of symptoms observed in our patient strongly supported correct diagnosis and effective treatment response. This case illustrates that syphilis could imitate lymphoma at multiple diagnostic levels (6, 16).

Beyond CTCL, the differential diagnosis of FDG-avid lymphadenopathy in concomitance with cutaneous manifestations includes a broad spectrum of infectious etiologies (such as tuberculosis, EBV- or CMV-associated reactive lymphadenitis, and bartonellosis), inflammatory conditions (such as sarcoidosis and IgG4-related disease), autoimmune diseases as well as non-lymphoma malignancies (5, 6, 13, 17, 18). Immunophenotyping often reveals a mixture of T- and B-lymphocytes without clear clonality, which can be interpreted as an early lymphoproliferative disorder unless molecular studies exclude it (6). FDG-PET-CT imaging, while highly sensitive for malignant lymphoma, lacks specificity; syphilitic lymphadenitis can demonstrate marked FDG uptake which may be challenging to distinguish from that of an aggressive lymphoma (4, 5, 8). In this context, incoherent findings - namely reactive histopathology without clonality and/or a disaccordance between extensive FDG uptake and the overall clinical presentation - should prompt diagnostic reassessment, including serologic testing for syphilis and, when feasible, spirochete-directed testing on tissue specimens. Thus, relying merely on histology or imaging may result in misdiagnosis.

Several case reports discuss this diagnostic challenge, as demonstrated in **Table 1**. Ohta et al. described generalized lymphadenopathy with pulmonary lesions mimicking lymphoma, ultimately diagnosed as syphilis after treponemal serology (4). Cerchione et al. reported a patient with FDG-PET-CT hypermetabolic nodes initially misdiagnosed as lymphoma until a late syphilis diagnosis was established (6). Yamashita et al. presented malignant syphilis in an HIV-positive patient, clinically and histologically resembling CTCL but lacking T-cell receptor clonality (7). Other reports document gastric syphilis initially suspected of adenocarcinoma or lymphoma (5), as well as skin manifestations initially diagnosed as drug eruption after antifungal therapy and primary cutaneous marginal zone lymphoma (9). Cervical lymph node involvement with pulmonary nodules has also been misinterpreted as lymphoma (8, 10).

**Figure 3:**
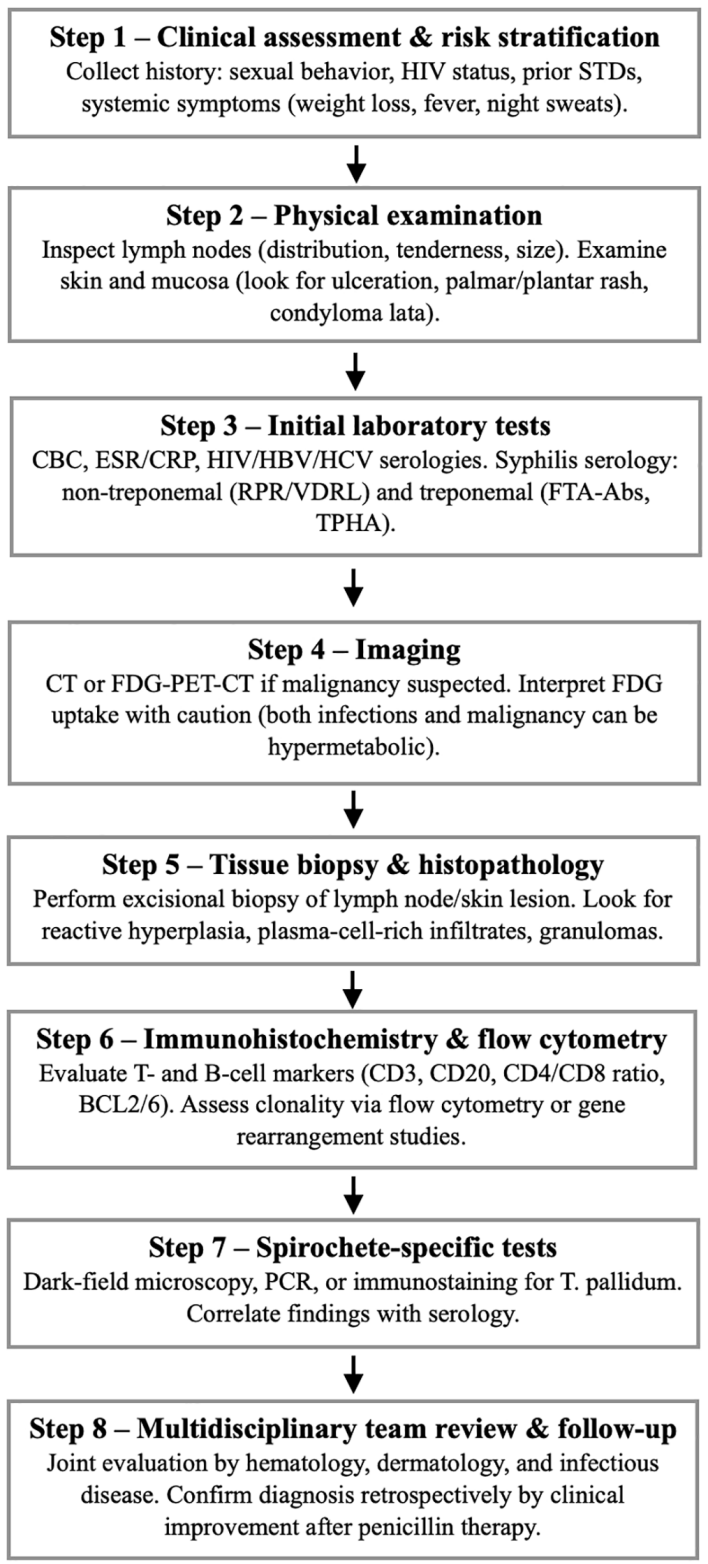
Proposed diagnostic workup of syphilis. STD, sexually transmitted diseases; CBC complete blood count; ESR, erythrocyte sedimentation rate; CRP, C, reactive protein; HIV, human immunodeficiency virus; HBV, hepatitis B virus; HCV, hepatitis C virus; RPR, rapid plasma reagin test; VDRL, Venereal Disease Research Laboratory test; FTA, Abs, fluorescent treponemal antibody absorption; TPHA, Treponema pallidum hemagglutination assay; CT, computed tomography, FDG, PET, CT fluorodeoxyglucose positron emission tomography combined with computed tomography; PCR, polymerase chain reaction.

Common diagnostic pitfalls across these cases include reliance on imaging suggestive of malignancy, nonspecific histology, and failure to consider syphilis serology early in the workup. Ultimately, serological testing, confirmatory immunohistochemistry or PCR for T. pallidum, history of high-risk sexual contact, and clinical resolution following penicillin therapy provide the final diagnostic confirmation. Our case adds to the already present scientific reports that polytypic plasma-cell infiltration without clonality and FDG-PET-CT findings of widespread hypermetabolism should always raise the suspicion of syphilis, particularly in young patients or those with risk factors. Based on our expertise and on previous scientific reports, we propose a diagnostic algorithm that may be of use to clinicians in their daily clinical practice (**Figure 3**).

Finally, from a therapeutic perspective, secondary syphilis, despite its potential to closely mimic hematologic malignancies, remains a condition with well-established and highly effective first-line treatment. Both the CDC and European IUSTI guidelines (13, 14) recommend benzathine penicillin G as the treatment of choice for early syphilis, including secondary syphilis, usually resulting in rapid clinical improvement and characteristic serologic response. Importantly, appropriate antimicrobial therapy is typically followed not only by resolution of cutaneous manifestations and systemic symptoms, but also by regression of lymphadenopathy and metabolic activity on FDG-PET-CT, thereby confirming the infectious nature of the process and preventing unnecessary oncologic interventions. While CDC guidelines emphasize serologic follow-up at 6 and 12 months after treatment (13), European IUSTI recommendations (14) recommend closer monitoring at 1, 3, 6, and 12 months, particularly in diagnostically complex cases or those presenting with high baseline titers, as illustrated in the present case.

## Conclusion

This case and the reviewed literature highlight the importance of considering syphilis in suspected hematologic and non-hematologic malignancies with inconclusive initial findings. When patients present with lymphadenopathy, systemic symptoms, and cutaneous lesions suspicious for lymphoma, syphilis should be included early in the differential diagnosis, especially in patients at risk. Serologic testing is widely available, hence its implementation may prevent invasive diagnostics and inappropriate therapies. Histopathology demonstrating reactive plasmacytic infiltration without clonality, or an FDG-PET-CT showing tracer uptake disproportionate to the clinical presentation, should prompt clinicians to consider syphilis. A multidisciplinary approach is essential to prevent delays in diagnosis and, eventually, reduce patient morbidity. Ultimately, systematic inclusion of syphilis in diagnostic workups can avoid misdiagnosis and unnecessary treatment, underscoring its relevance as an imitator of numerous disorders in modern hematology and internal medicine (1, 2, 12).

## Data Availability

The original contributions presented in the study are included in the article/supplementary material. Further inquiries can be directed to the corresponding author.
